# Financial toxicity burden among patients with cancer and cancer survivors in the UK: a cross-sectional study of prevalence and variation across recruitment pathways

**DOI:** 10.1136/bmjopen-2026-126799

**Published:** 2026-11-06

**Authors:** Tran Thu Ngan, Michael Donnelly, Damien Bennett, Ciaran O’Neill

**Affiliations:** 1 Centre for Public Health, Queen’s University Belfast, Belfast, UK; 2 Northern Ireland Cancer Registry, Belfast, UK

**Keywords:** Cancer Survivors, HEALTH ECONOMICS, Cost of Illness

## Abstract

**Abstract:**

**Objectives:**

To examine variation in financial toxicity (FT) according to recruitment pathway among patients with cancer and cancer survivors in the UK and identify sociodemographic and clinical factors associated with FT.

**Design:**

Cross-sectional online survey.

**Setting:**

Participants were recruited across the UK through an online research panel (Prolific) and community-oriented and support-oriented pathways, including cancer charities, patient support groups and social media.

**Participants:**

A total of 671 adults with a current or previous cancer diagnosis participated (497 recruited through the online research panel and 174 through community-oriented and support-oriented pathways).

**Primary and secondary outcome measures:**

FT was measured using the validated Comprehensive Score for Financial Toxicity (COST). FT was defined as a COST score <26, encompassing mild (14–25) and moderate/severe (0–13). Multivariable logistic regression was used to examine the association between recruitment pathway and FT after adjusting for age group, sex, occupation, personal income, cancer stage at diagnosis and treatment status.

**Results:**

In the study sample, 46.4% of participants experienced FT. FT prevalence was higher among participants recruited through community-oriented and support-oriented pathways than through the online research panel pathway (71.3% vs 37.6%, p<0.001). After adjustment for sociodemographic and clinical characteristics, participants recruited through community-oriented and support-oriented pathways had higher odds of FT (OR=2.83, 95% CI 1.69 to 4.75). Younger age, being unable to work due to illness and undergoing treatment were also independently associated with FT.

**Conclusions:**

In this cross-sectional study, observed FT burden differed substantially according to recruitment pathway. Participants recruited through community-oriented and support-oriented pathways had higher odds of FT than those recruited through an online research panel. These findings suggest that recruitment source should be considered when interpreting FT estimates and designing future studies, particularly where the aim is to understand financial vulnerability among people affected by cancer.

**STRENGTHS AND LIMITATIONS OF THIS STUDY**

- Participants were recruited through multiple pathways, enabling direct comparison between an online research panel and community-oriented and support-oriented recruitment approaches.
- Financial toxicity was measured using the validated Comprehensive Score for Financial Toxicity instrument.
- Multivariable analyses accounted for relevant sociodemographic and clinical characteristics.
- The cross-sectional design precludes conclusions regarding causal relationships between recruitment pathway and financial toxicity.
- The use of non-probability online sampling and self-reported data may limit the generalisability of the findings and introduce selection and reporting bias.

## Background

 Financial toxicity (FT) is increasingly recognised as an important consequence of cancer and its treatment. FT refers to the objective financial burden and subjective financial distress experienced by patients and their families as a result of cancer treatment and care.^1 2^ Although the concept was initially developed in the USA,^3^ growing evidence suggests that FT also affects patients in countries with publicly funded healthcare systems, including Canada, the UK and parts of Europe.^4–9^ In these countries, while healthcare services are largely free at the point-of-use, patients can still experience substantial indirect and non-medical costs, including loss of income, employment disruption, travel expenses, increased household costs or consume services not covered by third party payers such as complementary therapies or faster diagnostics. Previous UK research has reported that FT was associated with poorer health-related quality of life among patients with cancer and cancer survivors despite the UK’s publicly funded health system.^10^

Research on FT in the UK remains relatively limited compared with the USA, with much evidence relying on convenience sampling or single-source recruitment study designs.^11^ Recruitment pathway may influence reported FT burden because different approaches may engage patients with differing levels of clinical and financial vulnerability. Patients experiencing greater financial hardship or supportive care needs may be more likely to seek support from cancer charities, online support groups and community organisations. In contrast, participants recruited through online research panels may represent a broader group of patients with cancer and cancer survivors with differing levels of need. Consequently, estimates of FT may vary according to how participants are identified and recruited.

Cancer charities and support organisations play a vital role in providing practical, emotional and financial support to patients affected by cancer.^12 13^ These organisations may interact more closely with individuals experiencing unmet financial or supportive care needs that are not fully captured through routine healthcare systems or conventional research recruitment methods. Understanding differences between recruitment pathways will not only provide insights into methodological issues for FT research but will also provide novel information regarding patient groups who are more likely to engage with charitable and support services. This will have implications for FT screening, supportive care planning and future cancer survivorship research.

Previous studies which investigated FT among patients with cancer and cancer survivors in the UK and USA recruited exclusively through an online research platform only.^8 10^ However, little is known about how FT estimates differ by recruitment pathways, especially within the UK. This study aims to (1) estimate the prevalence and severity of FT in UK patients with cancer and cancer survivors; (2) examine differences in FT burden by recruitment pathways, specifically between online research platforms and community/support-oriented pathways and (3) identify sociodemographic and clinical factors associated with FT.

## Methods

This study was reported in accordance with the Strengthening the Reporting of Observational Studies in Epidemiology (STROBE) guidelines (online supplemental material).

### Study design and participants

A cross-sectional online survey was conducted among adults living in the UK who had received a cancer diagnosis. Participants were eligible if they were aged 18 years or older, resided in the UK and self-reported a current or previous diagnosis of cancer, regardless of treatment or clinical management status. Data collection was conducted between February and November 2025.

The survey was administered online using LimeSurvey (Hamburg, Germany). Participants were provided with study information electronically and completed an online consent form prior to participation.

### Recruitment pathways

Participants were recruited via a range of online routes, including Prolific (https://www.prolific.com/), an online participant recruitment platform widely used in health and social science research.^14–16^ Researchers can advertise studies to individuals meeting predefined eligibility criteria, and registered users can choose whether to participate. Participants were also recruited through paid advertisements on Facebook and Google, and posts shared by cancer charities, patient support groups and patient-led online communities.

For analysis, recruitment routes were grouped into two broad categories. The first consisted of participants recruited through Prolific and was referred to as the ‘online research panel’ pathway. The second group included participants recruited through cancer charities, patient support groups, online community sharing and paid social media/search advertisements, and was referred to as the ‘community and support-oriented’ pathway. This grouping was undertaken due to the small number of participants recruited through paid advertisements and the conceptual similarities between these recruitment approaches in reaching individuals engaged with cancer-related support or information networks.

### Variables and measurements

The main outcome—FT—was assessed using the COmprehensive Score for financial Toxicity (COST), a validated and widely used instrument consisting of 11 items.^17 18^ COST scores range from 0 to 44, with lower scores indicating greater FT. Consistent with previous studies and using established cut-off scores, participants were categorised as having no FT (COST ≥26), mild FT (COST 14–25) or moderate/severe FT (COST 0–13).^18^ For the binary outcome used in the logistic regression analyses, FT was defined as a COST score <26 and therefore included both mild and moderate/severe FT.

Information on sociodemographic and clinical characteristics was also collected. Sociodemographic variables included age, sex, ethnicity, employment status, personal annual income pre-tax, educational attainment and marital status. Clinical variables included cancer type, cancer stage at diagnosis and current treatment status (classified as currently receiving cancer treatment or having completed treatment). Age was categorised into three groups: <45 years, 45–64 years and ≥65 years.

### Statistical analysis

Descriptive statistics were used to summarise participant characteristics and the prevalence of FT across recruitment pathways. Categorical variables were presented as frequencies and percentages. Comparisons between groups were conducted using χ^2^ tests.

Separate analyses were conducted for each recruitment pathway to examine sociodemographic and clinical patterning of FT within different participant groups. Univariable and multivariable logistic regression models were used to examine factors associated with FT. Variables included in multivariable analyses were selected based on existing literature and clinical relevance. The multivariable models included recruitment pathway as the primary explanatory variable, adjusting for age group, sex, occupation, personal income, cancer stage at diagnosis and treatment status. Missing data were handled using complete-case analysis, with participants who had missing values for any variable included in the model excluded from the analysis. No imputation was performed.

Adjusted ORs and 95% CIs were reported. Statistical significance was set at p<0.05. All analyses were conducted using Stata V.19.0.

### Patient and public involvement

Patients and members of the public were actively involved throughout the development and conduct of this research. The study concept and survey design were presented to the Northern Ireland Cancer Consumer Forum, and feedback from patient representatives informed the study design, participant-facing materials and recruitment strategy. A study Steering Committee, including patient representatives from all four UK nations, was established to provide ongoing oversight of the project. In addition, a patient and public involvement (PPI) group comprising patient representatives from across the UK was formed to advise on study conduct, recruitment and participant engagement. Members of the PPI group contributed to promoting the study through their networks and patient organisations and will also be involved in disseminating the study findings to patient communities and the wider public.

## Results

### Participant characteristics by recruitment pathway

A total of 671 participants completed the survey and were included in the analysis. Of these, 497 (74%) participants were recruited through the online research panel pathway (Prolific), while 174 (26%) were recruited through community-oriented and support-oriented pathways, including cancer charities, patient support groups and social media/community dissemination.

Participant characteristics by recruitment pathway are presented in table 1. The mean age of participants was 55 years (SD=13 years, range=19–85 years). Female participants accounted for 72%; 40% participants had breast cancer (online supplemental table S1); 67% were married and 80% had at least high school diploma. The majority of participants were white (94%). Regarding occupation, 32% participants worked full-time, 28% were part-time or self-employed and 17% were retired. Regarding treatment status, 66% of participants had completed all treatments.

**Table 1:** Participant sociodemographic and clinical characteristics by recruitment pathway

	Total, n (%)	Community and support-oriented, n (%)	Online research panel, n (%)	P value
Total	671 (100)	174 (25.9)	497 (74.1)	
Age, mean (SD)	55 (13)	54 (12)	55 (13)	0.43
Age group				
<45 years old	152 (22.8)	40 (23.4)	112 (22.5)	0.20
45–64 years old	354 (53.0)	98 (57.3)	256 (51.5)	
65+ years old	162 (24.3)	33 (19.3)	129 (26.0)	
Sex				
Male	187 (28.1)	38 (22.2)	149 (30.2)	0.05
Female	478 (71.9)	133 (77.8)	345 (69.8)	
Race/ethnicity				
White	629 (94.2)	164 (95.9)	465 (93.6)	0.75
Others	42 (6.3)	10 (5.7)	32 (6.4)	
Education				
Postgraduate degree and above	132 (19.7)	50 (28.7)	82 (16.5)	<0.001
Undergraduate degree	225 (33.5)	54 (31.0)	171 (34.4)	
A-level/HND or equivalent	178 (26.5)	32 (18.4)	146 (29.4)	
School certificate/GCSE/O-level/NVQ or equivalent	124 (18.5)	32 (18.4)	92 (18.5)	
Other	12 (1.7)	6 (3.4)	6 (1.2)	
Marital status				
Single	117 (17.5)	23 (13.5)	94 (18.9)	0.14
Married/cohabiting	447 (66.9)	121 (70.8)	326 (65.6)	
Others (divorced, separated, widowed)	104 (15.6)	27 (15.8)	77 (15.5)	
Occupation				
Employed full-time	207 (32.2)	32 (19.6)	175 (36.5)	<0.001
Employed part-time or self-employed	182 (28.3)	42 (25.8)	140 (29.2)	
Unemployed/not working	62 (9.6)	12 (7.4)	50 (10.4)	
Retired	106 (16.5)	29 (17.8)	77 (16.0)	
Disabled/too ill to work	86 (13.4)	48 (29.4)	38 (7.9)	
Personal annual income				
<£12 570	114 (18.0)	23 (15.0)	91 (19.0)	0.07
£12 570–£25 000	192 (30.4)	37 (24.2)	155 (32.4)	
£25 001–£50 000	227 (35.9)	63 (41.2)	164 (34.2)	
More than £50 000	99 (15.7)	30 (19.6)	69 (14.4)	
Stage at diagnosis				
Early stages	367 (65.7)	73 (46.5)	294 (73.1)	<0.001
Late stages	192 (34.3)	84 (53.5)	108 (26.9)	
Treatment status				
Undergoing any type of treatment	226 (33.7)	105 (60.3)	121 (24.3)	<0.001
Completed all treatment	445 (66.3)	69 (39.7)	376 (75.7)	

GCSE, General Certificate of Secondary Education; HND, Higher National Diploma; NVQ, National Vocational Qualification.

Sociodemographic characteristics were broadly similar between the two recruitment pathways with no significant differences observed in age, sex, marital status, household income or ethnicity. However, occupational status differed significantly between groups. Participants recruited through community-oriented and support-oriented pathways were more likely to report being disabled or too ill to work and were less likely to be in full-time employment compared with those recruited through the online research panel pathway. Educational attainment also differed, with a higher proportion of participants in the community-oriented and support-oriented pathway reporting higher levels of education.

Several clinical characteristics differed significantly between recruitment pathways. Participants recruited through community-oriented and support-oriented pathways were more likely to report later-stage disease (54% vs 27%, p<0.001) and were substantially more likely to be undergoing cancer treatment at the time of the survey compared with participants recruited through the online research panel pathway (60% vs 24%, p<0.001). Cancer type also differed between recruitment pathways (online supplemental table S1).

### Prevalence and severity of FT

In the study sample, 46.4% of participants faced FT. FT prevalence differed markedly according to recruitment pathway. Among participants recruited through the online research panel pathway, 37.6% experienced FT, compared with 71.3% of those recruited through community-oriented and support-oriented pathways (χ^2^ test, p<0.001).

Differences in FT severity distribution between recruitment pathways were also statistically significant (χ^2^ test, p<0.001) (figure 1). Among participants recruited through community-oriented and support-oriented pathways, 32.2% experienced moderate/severe FT compared with only 11.1% in the online research panel pathway. In contrast, participants recruited through the online research panel pathway were much more likely to report no FT (62.4% vs 28.7%).

**Figure 1:**
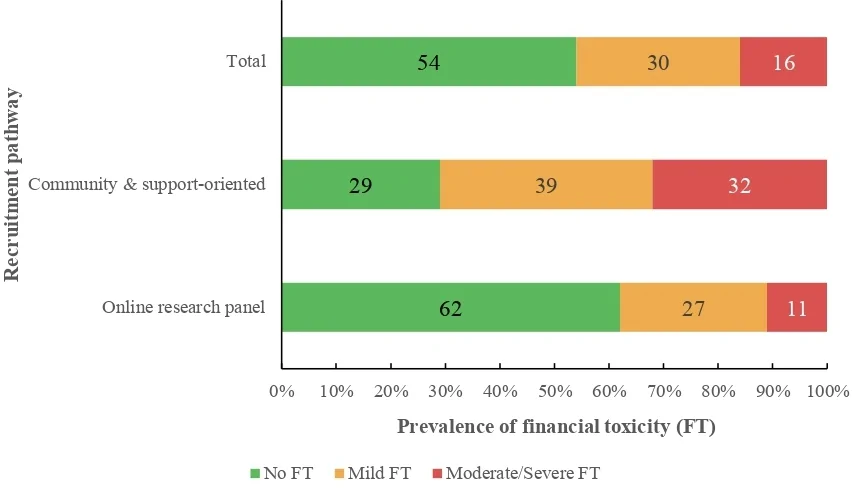
Distribution of financial toxicity severity by recruitment pathway.

### Factors associated with FT within recruitment pathways

In analyses stratified by recruitment pathway, younger participants and those currently undergoing treatment consistently reported higher levels of FT across both recruitment pathways (table 2).

**Table 2:** Financial toxicity prevalence according to sociodemographic and clinical characteristics within each recruitment pathway

	**Community/support pathway**		**Online research panel**	
	Participants with FT, n (%)	P value*	Participants with FT, n (%)	P value*
Total	124 (71.3)		187 (37.6)	
Age group				
<45 years old	37 (92.5)	0.001	60 (53.6)	<0.001
45–64 years old	68 (69.4)		103 (40.2)	
65+ years old	18 (54.5)		24 (18.6)	
Sex				
Male	22 (57.9)	0.03	48 (32.2)	0.10
Female	101 (75.9)		138 (40.0)	
Education				
Postgraduate degree or above	34 (68.0)	0.80	25 (30.5)	0.23
Undergraduate degree (BA/BSc/other)	38 (70.4)		66 (38.6)	
A-level/HND or equivalent	25 (78.1)		52 (35.6)	
Others	23 (71.1)		44 (44.9)	
Marital status				
Single	17 (73.9)	0.93	41 (43.6)	0.40
Married/cohabiting	86 (71.1)		117 (35.9)	
Others (divorced/separated/widowed)	20 (74.1)		29 (37.7)	
Race/ethnicity				
White	117 (71.3)	0.93	170 (36.6)	0.06
Others	7 (70.0)		17 (53.1)	
Personal annual income				
<£12 570	18 (78.3)	0.93	41 (45.1)	0.46
£12 570–£25 000	26 (70.3)		57 (36.8)	
£25 001–£50 000	46 (73.0)		59 (36.0)	
More than £50 000	22 (73.3)		24 (34.8)	
Occupation				
Employed full-time	20 (62.5)	0.01	52 (29.7)	<0.001
Employed part-time or self-employed	31 (73.8)		57 (40.7)	
Unemployed/not working	8 (66.7)		30 (60.0)	
Retired	15 (51.7)		12 (15.6)	
Disabled/too ill to work	42 (87.5)		31 (81.6)	
Stage of cancer at diagnosis				
Early stages	50 (68.5)	0.69	106 (36.1)	0.13
Late stages	60 (71.4)		48 (44.4)	
Treatment status				
Undergoing any type of treatment	83 (79.0)	0.01	58 (47.9)	0.01
Completed all treatment	41 (59.4)		129 (34.3)	

* χ2 test comparing FT prevalence across categories within each recruitment pathway.

FT, financial toxicity; HND, Higher National Diploma.

Within the online research panel pathway, FT prevalence was 53.6% among participants aged below 45 years, compared with 40.2% among those aged 45–64 years and 18.6% among those aged 65 years or older (χ^2^ test, p<0.001). In the community-oriented and support-oriented pathway, corresponding FT prevalence was 92.5%, 69.4% and 54.5%, respectively (χ^2^ test, p=0.001).

Participants currently undergoing treatment reported much higher FT than those who had completed treatment for both recruitment pathways. In the online research panel pathway, 47.9% of those undergoing treatment reported FT compared with 34.3% of those who had completed treatment (χ^2^ test, p=0.01). In the community-oriented and support-oriented pathway, 79.0% of those undergoing treatment reported FT compared with 59.4% who had completed treatment (χ^2^ test, p=0.01).

### Pooled multivariable analyses

In pooled multivariable logistic regression analyses, recruitment pathway remained independently associated with FT after adjustment for age, sex, occupational status, personal annual income pre-tax, self-reported stage of cancer at diagnosis and treatment status (table 3). Participants recruited through community-oriented and support-oriented pathways had substantially higher odds of FT compared with those recruited through the online research panel pathway (OR=2.83, 95% CI 1.39 to 4.75).

**Table 3:** Pooled multivariable logistic regression analysis of factors associated with financial toxicity

	Adjusted ORs (aORs)*	95% CI
Recruitment pathways		
Online research panel^ref^	1.00	
Community and support-oriented	2.83†	(1.69 to 4.75)
Age group		
<45 years old^ref^	1.00	
45–64 years old	0.36‡	(0.22 to 0.61)
65+ years old	0.21‡	(0.11 to 0.41)
Sex		
Male^ref^	1.00	
Female	1.26	(0.78 to 2.04)
Occupation		
Employed full-time^ref^	1.00	
Employed part-time or self-employed	1.47	(0.89 to 2.44)
Unemployed/not working	2.64†	(1.17 to 5.96)
Retired	0.74	(0.36 to 1.54)
Disabled/too ill to work	7.37‡	(3.26 to 16.69)
Treatment status		
Completed all treatment^ref^	1.00	
Undergoing any type of treatment	1.70†	(1.08 to 2.68)
Personal income		
<£12 570^ref^	1.00	
£12 570–£25 000	0.86	(0.45 to 1.65)
£25 001–£50 000	0.94	(0.49 to 1.82)
More than £50 000	0.78	(0.36 to 1.69)
Stage at diagnosis		
Early stages^ref^	1.00	
Late stages	1.05	(0.67 to 1.65)

* Exponentiated coefficients.

† p <0.05 versus reference group.

‡ p <0.001 versus reference group.

ref, reference group.

FT was more commonly reported among younger participants. Compared with participants aged below 45 years, those aged 45–64 years (OR=0.36, 95% CI 0.22 to 0.61) and those aged 65 years or older (OR=0.21, 95% CI 0.11 to 0.41) had significantly lower odds of FT. Participants who were currently undergoing treatment had higher odds of FT than those who had completed treatment (OR=1.70, 95% CI 1.08 to 2.68).

Occupational status was strongly associated with FT. Compared with participants in full-time employment, unemployed participants (OR=2.64, 95% CI 1.17 to 5.96) and those who were disabled or too ill to work (OR=7.37, 95% CI 3.26 to 16.69) had substantially higher odds of FT.

Findings were consistent in ordered logistic regression analyses examining FT severity categories, with recruitment pathway remaining significantly associated with greater FT severity after adjustment for sociodemographic and clinical characteristics (online supplemental table S2).

## Discussion

In this study, nearly one in two UK patients with cancer and cancer survivors experienced FT, with approximately one in six reporting moderate to severe FT, despite receiving care within a publicly funded healthcare system. FT burden showed marked differences according to recruitment pathway. Participants recruited through community-oriented and support-oriented pathways reported significantly higher FT and greater levels of FT severity than those recruited through an online research panel. Higher levels of FT were reported by participants undergoing treatment, those with later-stage disease and those who were disabled or too ill to work. Importantly, the recruitment pathway remained independently associated with FT after adjustment for key sociodemographic and clinical factors, suggesting that different recruitment approaches may engage populations with differing levels of clinical and financial vulnerability.

### Prevalence and severity of FT

Our findings add to growing evidence that FT remains a substantial issue even in countries with publicly funded healthcare systems.^4–9^ Although the National Health Service reduces direct medical costs, patients may still experience considerable financial burden related to reduced income, employment disruption, transport costs, increased household expenses and other indirect costs associated with cancer and its treatment.^11 19–21^ The overall prevalence of FT observed in this study (46.4%) was broadly consistent with previous cancer research. Parker *et al* reported FT in 42% of Australian patients with haematological malignancies receiving care within a publicly funded healthcare system.^22^ A systematic review and meta-analysis of patients with breast cancer reported a pooled FT prevalence of 35.3% in high-income countries,^23^ while a broader meta-analysis of cancer survivors estimated a pooled prevalence of 45%.^24^ Direct comparisons should nevertheless be interpreted cautiously because of differences in cancer populations, recruitment approaches and definitions and measures of FT across studies.

More specifically, FT prevalence among participants recruited through Prolific in the current study (37.6%) was slightly higher than that observed in our earlier UK study conducted in 2023 using the same recruitment platform (33%).^8^ Although the studies involved different participant samples and are not directly comparable, this pattern may reflect broader economic pressures during the study period, including rising living costs and ongoing financial pressures affecting households in the UK.

A key finding of this study was the substantial difference in observed FT burden across recruitment pathways. Participants recruited through community-oriented and support-oriented pathways reported considerably higher FT prevalence and severity than those recruited through the online research panel pathway. These differences appeared partly related to clinical vulnerability, as participants recruited through community-oriented and support-oriented pathways were more likely to have later-stage disease and be currently undergoing treatment. Participants recruited via these pathways also had higher levels of educational attainment, which is consistent with previous research suggesting that engagement with patient support groups and supportive care services may be more common among individuals who are closer to diagnosis or treatment and those with higher levels of education.^25–28^ However, recruitment pathway remained independently associated with FT after adjustment for treatment status and other covariates. One possible explanation is that patients experiencing greater financial and supportive care needs may be more likely to engage with cancer charities, support groups and online patient communities.^28 29^ In contrast, online research panels may capture a broader group of patients with cancer and cancer survivors with differing levels of need. Together, these findings suggest that FT reports and estimates may be shaped not only by patient characteristics, but also by how participants are identified and recruited.

The findings also highlight the potential role of cancer charities and support organisations in identifying and supporting patients experiencing financial hardship. Patients recruited through these pathways demonstrated high levels of FT and greater clinical vulnerability, suggesting that support organisations may interact closely with individuals experiencing unmet supportive care needs. Previous studies have highlighted the importance of financial counselling, welfare advice, employment support and psychosocial services in reducing the impact of FT.^30–32^ Strengthening links between healthcare providers and support organisations may therefore help improve identification of patients at risk of FT and facilitate earlier access to supportive interventions.

### Factors associated with FT in the UK

Consistent with previous literature, younger people, participants currently undergoing treatment and individuals unable to work due to illness reported higher levels of FT.^2 9 11^ Younger patients may experience greater employment and financial disruption and may also have reduced financial assets, reserves or savings compared with older individuals. Ongoing treatment may increase both direct and indirect financial pressures through reduced capacity to work, higher transport costs and additional day-to-day expenses associated with cancer care. Participants who were disabled or too ill to work represented a particularly vulnerable group, with substantially higher odds of FT compared with those in full-time employment. These findings reinforce evidence that FT is closely linked to employment disruption and reduced earning capacity during and after cancer treatment.^2 9^

The strong association between FT and inability to work is particularly important within the UK context, where healthcare is largely free at the point of use. As a result, cancer-related financial hardship may be less visible and is often assumed to be less problematic than in healthcare systems with substantial out-of-pocket medical costs. Unlike healthcare systems where FT may be driven predominantly by direct medical costs, FT in the UK may arise more from loss of income, reduced productivity and broader socioeconomic consequences of cancer and its treatment.^11 19^ Patients undergoing treatment may experience prolonged sickness absence, reduced working hours or difficulties returning to employment, all of which may contribute to ongoing financial strain. Previous studies have similarly highlighted employment disruption as a major contributor to FT and poorer survivorship outcomes among patients with cancer.^32 33^ These findings suggest that reducing FT may require not only financial support, but also interventions aimed at maintaining employment, supporting return-to-work and improving access to welfare and supportive care services during and after treatment.

The lower FT burden observed among participants who had completed treatment may suggest that financial difficulties improve for some individuals following treatment completion. However, FT is unlikely to be a uniform experience. While some patients may experience temporary financial strain associated with treatment-related costs and employment disruption, others may face more persistent financial consequences due to prolonged work absence, reduced earning capacity, depletion of savings or accumulated debt. Previous research has highlighted that the financial impact of cancer can extend well beyond the treatment period for some survivors, indicating the need for longer-term support and follow-up.^32 34^

### Strengths and limitations

This study has several strengths. To the best of our knowledge, this is the first study to investigate FT prevalence among UK patients with cancer and cancer survivors across different recruitment pathways. It included a relatively large UK sample and incorporated multiple recruitment pathways, allowing direct comparison of FT burden across different recruitment contexts. The use of multiple recruitment pathways also enabled inclusion of patients with differing levels of clinical and financial vulnerability, reducing reliance on a single recruitment approach. The study also examined FT severity in addition to binary FT measures.

However, several limitations should be considered. The study used non-probability online sampling methods and relied on self-reported data, which may introduce selection and reporting biases. The cross-sectional design also limits causal interpretation. In addition, the sample consisted predominantly of White participants, limiting exploration of potential ethnic differences in FT. Although participants with various cancer types were included, subgroup analyses by cancer site were not possible because of small sample sizes within individual cancer groups. Area-level socioeconomic deprivation was not measured; therefore, the study could not examine inequalities according to measures such as the Index of Multiple Deprivation. Neither recruitment pathway should be considered nationally representative of the UK cancer population. Nevertheless, the findings demonstrate that recruitment approach can substantially influence observed FT estimates and may shape which patient groups are represented in FT research.

## Conclusions

Overall, this study found that FT remains common among UK patients with cancer and cancer survivors and that FT burden differs substantially and significantly across recruitment pathways. Community-oriented and support-oriented pathway recruitment may engage patients with particularly high levels of financial and clinical vulnerability. These findings suggest that reliance on a single recruitment source may provide an incomplete picture of FT burden, highlighting the importance of diverse recruitment and engagement strategies in FT research, needs assessment and supportive care planning.

## Supplementary material

10.1136/bmjopen-2026-126799 online supplemental file 1

10.1136/bmjopen-2026-126799 online supplemental file 2

## Data Availability

All data relevant to the study are included in the article or uploaded as supplementary information.

## References

[1] Zafar SY, Peppercorn JM, Schrag D (2013). The financial toxicity of cancer treatment: a pilot study assessing out-of-pocket expenses and the insured cancer patient’s experience. Oncologist.

[2] Zafar SY, Abernethy AP (2013). Financial toxicity, Part I: a new name for a growing problem. Oncology (Williston Park).

[3] Witte J, Mehlis K, Surmann B (2019). Methods for measuring financial toxicity after cancer diagnosis and treatment: a systematic review and its implications. Ann Oncol.

[4] Honda K, Gyawali B, Ando M (2019). Prospective Survey of Financial Toxicity Measured by the Comprehensive Score for Financial Toxicity in Japanese Patients With Cancer. J Glob Oncol.

[5] Perrone F, Jommi C, Di Maio M (2016). The association of financial difficulties with clinical outcomes in cancer patients: secondary analysis of 16 academic prospective clinical trials conducted in Italy. *Ann Oncol*.

[6] Fabian A, Domschikowski J, Greiner W (2022). Financial toxicity in cancer patients treated with radiotherapy in Germany-a cross-sectional study. Strahlenther Onkol.

[7] Fitch MI, Longo CJ (2021). Emerging Understanding About the Impact of Financial Toxicity Related to Cancer: Canadian Perspectives. Semin Oncol Nurs.

[8] Ngan TT, Tonorezos E, Donnelly M (2025). Financial toxicity amongst cancer patients and survivors: a comparative study of the United Kingdom and United States. *Support Care Cancer*.

[9] Vancoppenolle J, Franzen N, Azarang L (2025). Financial toxicity and socioeconomic impact of cancer in Europe. ESMO Open.

[10] Ngan TT, Tonorezos E, Donnelly M (2025). Impact of Financial Toxicity on the Health-Related Quality of Life and Financial Well-Being of Cancer Patients and Survivors: A Comparative Study of the United Kingdom and United States. Cancer Med.

[11] Ngan TT, Tien TH, Donnelly M (2023). Financial toxicity among cancer patients, survivors and their families in the United Kingdom: a scoping review. J Public Health (Oxf).

[12] Baker BS, Marshall J, Hoffman CJ (2019). Evaluation of a charity-led secondary breast cancer support day: a model of survivorship care. Ecancermedicalscience.

[13] Leedham K, Platt K (1998). Community cancer support – the Neil Cliffe Cancer Care charity approach to supportive care. Int J Palliat Nurs.

[14] Boyd P, Murray AB, Hyams T (2022). Neuroticism, cancer mortality salience, and physician avoidance in cancer survivors: Proximity of treatment matters. Psychooncology.

[15] Yuen EYN, Wilson CJ (2021). The Relationship between Cancer Caregiver Burden and Psychological Outcomes: The Moderating Role of Social Connectedness. *Current Oncology*.

[16] Peer E, Brandimarte L, Samat S (2017). Beyond the Turk: Alternative platforms for crowdsourcing behavioral research. J Exp Soc Psychol.

[17] de Souza JA, Yap BJ, Hlubocky FJ (2014). The development of a financial toxicity patient-reported outcome in cancer: The COST measure. Cancer.

[18] de Souza JA, Yap BJ, Wroblewski K (2017). Measuring financial toxicity as a clinically relevant patient-reported outcome: The validation of the COmprehensive Score for financial Toxicity (COST). Cancer.

[19] Longo CJ, Fitch MI, Banfield L (2020). Financial toxicity associated with a cancer diagnosis in publicly funded healthcare countries: a systematic review. *Support Care Cancer*.

[20] Macmillian Cancer Support (2017). No small change: time to act on the financial impact of cancer.

[21] Davies SV, Hayes DA, Macmillian Cancer Support (2013). Cancer’s hidden price tag: revealling the costs behind the illness.

[22] Parker C, Ayton D, Zomer E (2022). Do patients with haematological malignancies suffer financial burden? A cross-sectional study of patients seeking care through a publicly funded healthcare system. Leuk Res.

[23] Ehsan AN, Wu CA, Minasian A (2023). Financial Toxicity Among Patients With Breast Cancer Worldwide: A Systematic Review and Meta-analysis. JAMA Netw Open.

[24] Jiang H, Lyu J, Mou W (2023). Prevalence and risk factors of self-reported financial toxicity in cancer survivors: A systematic review and meta-analyses. J Psychosoc Oncol.

[25] Berglund G, Bolund C, Gustafsson UL (1997). Is the wish to participate in a cancer rehabilitation program an indicator of the need? Comparisons of participants and non-participants in a randomized study. Psychooncology.

[26] Katz D, Koppie TM, Wu D (2002). Sociodemographic characteristics and health related quality of life in men attending prostate cancer support groups. J Urol.

[27] Stevens MJ, Duttlinger JE (1998). Correlates of participation in a breast cancer support group. J Psychosom Res.

[28] Taylor SE, Falke RL, Shoptaw SJ (1986). Social *support, support groups, and the cancer p*atient.

[29] Carlsson C, Baigi A, Killander D (2005). Motives for becoming and remaining member of patient associations: a study of 1,810 Swedish individuals with cancer associations. *Support Care Cancer*.

[30] Carrera PM, Kantarjian HM, Blinder VS (2018). The financial burden and distress of patients with cancer: Understanding and stepping-up action on the financial toxicity of cancer treatment. *CA Cancer J Clin*.

[31] Fitch M, Longo CJ (2018). Exploring the impact of out-of-pocket costs on the quality of life of Canadian cancer patients. J Psychosoc Oncol.

[32] Gordon LG, Merollini KMD, Lowe A (2017). A Systematic Review of Financial Toxicity Among Cancer Survivors: We Can’t Pay the Co-Pay. *Patient*.

[33] de Boer AGEM, Taskila T, Ojajärvi A (2009). Cancer survivors and unemployment: a meta-analysis and meta-regression. JAMA.

[34] Chan RJ, Gordon LG, Tan CJ (2019). Relationships Between Financial Toxicity and Symptom Burden in Cancer Survivors: A Systematic Review. J Pain Symptom Manage.

