# Supplementary material for: Financial toxicity burden among patients with cancer and cancer survivors in the UK: a cross-sectional study of prevalence and variation across recruitment pathways
Source: BMJ Open. 2026 Nov 6;16(11):e126799. doi: 10.1136/bmjopen-2026-126799 (PMC13756267; doi:10.1136/bmjopen-2026-126799)
Supplement: Supplementary file 1 [file bmjopen-2026-126799.reviewer_comments.pdf]

PEER REVIEW HISTORY

BMJ Open publishes all reviews undertaken for accepted manuscripts. Reviewers are asked to complete a checklist review form and are provided with free text boxes to elaborate on their assessment. These free text comments are reproduced below.

ARTICLE DETAILS

Title (Provisional)

Financial toxicity burden among cancer patients and survivors in the United Kingdom: a cross-sectional study of prevalence and variation across recruitment pathways

Authors

Ngan, Tran Thu; Donnelly, Michael; Bennett, Damien; O'Neill, Ciaran

---

VERSION 1 - REVIEW

**Reviewer**	**1**
**Name**	**Bucholc, Jessica**
**Affiliation**	**Deakin University, Health Economics**
**Date**	**07-Oct-2026**
**COI**	**None**

Dear Authors, this is a very well-written paper, thank you for the ease of reading it. The study is clearly presented and the results support the overall conclusion, I recommend minor revision only. All my suggestions do not affect the quality of the study.

Suggestions:

1. The abstract aims d not fully match the aims stated in the introduction of the paper. Consider revising.

2. Abstract clnclusion places emphasis of the support organisations, while the manuscript consludion focuses of differences by recruitment pathway and importance of recruitment strategies. Consider aligning.

3. You have listed in the limitations that the sample cannot be nationally representative. Consider noting more clearly that the overall FT prevalence (46.4%) reflects the study sample rather than UK pop.

4. Consider providing a short explanation of how missing data was handled.

**Reviewer**	**2**

**Name**	**Cooper, Keith**
**Affiliation**	**University of Southampton**
**Date**	**20-Oct-2026**
**COI**	**None**

This is a well written study. I would have liked to see financial toxicity clearly defined. I was unsure whether FT referred to those with mild, moderate and severe FT or just moderate and severe FT, so perhaps this could be made clear.

I would have liked to see how FT related to inequality, such as index of multiple deprivation but this doesn't appear to have been included which, to me, is a limitation. Perhaps this could be commented on in the discussion section.

Perhaps you could also add how the proportion with FT in this study varied from other studies in the UK and elsewhere for cancer or other morbidities.

The strengths and limitations of this study could be improved by emphasising the key takeaway points of the study.

VERSION 1 - AUTHOR RESPONSE

REVIEWER: 1
Ms. Jessica Bucholc, Deakin University

Comments to the Author:
Dear Authors, this is a very well-written paper, thank you for the ease of reading it. The study is clearly presented and the results support the overall conclusion, I recommend minor revision only. All my suggestions do not affect the quality of the study.

Suggestions:
1. The abstract aims do not fully match the aims stated in the introduction of the paper. Consider revising.

> **Response:** We have revised the aim stated in the Abstract to ensure consistency with the study aims outlined in the Introduction. The revised aim reads as follows:
>
> "To examine variation in financial toxicity (FT) according to recruitment pathway among cancer patients and survivors in the United Kingdom (UK) and identify sociodemographic and clinical factors associated with FT."

2. Abstract conclusion places emphasis of the support organisations, while the manuscript conclusion focuses of differences by recruitment pathway and importance of recruitment strategies. Consider aligning.

> **Response:** We have revised the Abstract Conclusion to align more closely with the manuscript Conclusion, shifting the emphasis from support organisations to differences in financial toxicity by recruitment pathway and the methodological importance of recruitment strategies in research. The revised conclusion reads as follows:

“In this cross-sectional study, observed FT burden differed substantially according to recruitment pathway. Participants recruited through community- and support-oriented pathways had higher odds of FT than those recruited through an online research panel. These findings suggest that recruitment source should be considered when interpreting FT estimates and designing future studies, particularly where the aim is to understand financial vulnerability among people affected by cancer.”

3. You have listed in the limitations that the sample cannot be nationally representative. Consider noting more clearly that the overall FT prevalence (46.4%) reflects the study sample rather than UK pop.

Response: We have revised the Abstract Results as well as the results section in the paper to clarify that the reported FT prevalence (46.4%) refers specifically to the study sample and should not be interpreted as representative of the wider UK population. The revised sentence reads: “In the study sample, 46.4% of participants experienced FT.”

4. Consider providing a short explanation of how missing data was handled.

Response: Thank you for your comment. We have added a brief explanation of how missing data were handled in the Methods section, as following:

“Missing data were handled using complete-case analysis for the multivariable logistic regression models, with participants who had missing values for any variable included in the model excluded from the analysis. No imputation was performed.”

REVIEWER: 2
Dr. Keith Cooper, University of Southampton

Comments to the Author:

This is a well written study. I would have liked to see financial toxicity clearly defined. I was unsure whether FT referred to those with mild, moderate and severe FT or just moderate and severe FT, so perhaps this could be made clear.

Response: Thank you for your positive feedback. We agree that the definition of financial toxicity (FT) should be clearer. We have therefore revised the Methods section to explicitly define FT, including the cut-off scores used to classify its severity. We have also clarified that, for the logistic regression analyses, FT includes participants experiencing either mild or moderate/severe FT. The following text has been added to the Methods section:

“Consistent with previous studies and using established cut-off scores, participants were categorised as having no FT (COST>=26), mild FT (COST 14-25), or moderate/severe FT (COST 0-13) [18]). For the binary outcome used in the logistic regression analyses, FT was defined as a COST score <26 and therefore included both mild and moderate/severe FT. ”

I would have liked to see how FT related to inequality, such as index of multiple deprivation but this doesn't appear to have been included which, to me, is a limitation. Perhaps this could be commented on in the discussion section.

Response: We agree that examining the relationship between financial toxicity (FT) and socioeconomic deprivation would provide further insight into potential inequalities in FT. As area-level socioeconomic deprivation was not assessed in this study, we have acknowledged this limitation in the Discussion section by adding the following statement:

“Area-level socioeconomic deprivation was not measured; therefore, the study could not examine inequalities according to measures such as the Index of Multiple Deprivation.”

Perhaps you could also add how the proportion with FT in this study varied from other studies in the UK and elsewhere for cancer or other morbidities.

Response: Thank you for this helpful suggestion. The Discussion previously noted that the prevalence observed in our study was consistent with previous UK and international research, but we agree that the comparison was not sufficiently explicit. We have therefore revised the Discussion to provide quantitative comparisons with previous cancer studies.

“The overall prevalence of FT observed in this study (46.4%) was broadly consistent with previous cancer research. Parker et al. reported FT in 42% of Australian patients with haematological malignancies receiving care within a publicly funded healthcare system [22]. A systematic review and meta-analysis of patients with breast cancer reported a pooled FT prevalence of 35.3% in high-income countries [23], while a broader meta-analysis of cancer survivors estimated a pooled prevalence of 45% [24]. Direct comparisons should nevertheless be interpreted cautiously because of differences in cancer populations, recruitment approaches and definitions and measures of FT across studies.

More specifically, FT prevalence among participants recruited through Prolific in the current study (37.6%) was slightly higher than that observed in our earlier UK study conducted in 2023 using the same recruitment platform (33%) [8].”

The strengths and limitations of this study could be improved by emphasising the key takeaway points of the study.

Response: Thank you for this suggestion. We have revised the Strengths and limitations section to more clearly emphasise the methodological features that are most important for interpreting the study, including the use of multiple recruitment pathways, the validated COST instrument, adjustment for relevant covariates, the cross-sectional design and the implications of non-probability sampling for generalisability. In accordance with the Editor's guidance that this summary box should relate specifically to study methods and should not summarise the study's results or expected impact, we have retained the principal study findings and implications in the Discussion and Conclusions sections.

The revised ‘Strengths and limitations of this study’ section is as following:

- Participants were recruited through multiple pathways, enabling direct comparison between an online research panel and community- and support-oriented recruitment approaches.
- Financial toxicity was measured using the validated Comprehensive Score for Financial Toxicity (COST) instrument.
- Multivariable analyses accounted for relevant sociodemographic and clinical characteristics.
- The cross-sectional design precludes conclusions regarding causal relationships between recruitment pathway and financial toxicity.
- The use of non-probability online sampling and self-reported data may limit the generalisability of the findings and introduce selection and reporting bias.
