# Supplementary material for: Financial toxicity burden among patients with cancer and cancer survivors in the UK: a cross-sectional study of prevalence and variation across recruitment pathways
Source: BMJ Open. 2026 Nov 6;16(11):e126799. doi: 10.1136/bmjopen-2026-126799 (PMC13756267; doi:10.1136/bmjopen-2026-126799)
Supplement: Supplementary file 3 [file bmjopen-16-11-s002.docx]

**Supplementary materials**

**Financial toxicity among cancer patients and survivors in the United Kingdom: prevalence and variation across recruitment pathways**

Tran Thu Ngan^1 *^, Michael Donnelly^1^, Damien Bennett^2^, Ciaran O’Neill^1^

*^1^ Centre for Public Health, Queen’s University Belfast, Belfast, United Kingdom*

*^2^ Northern Ireland Cancer Registry, Queen’s University Belfast, Belfast, United Kingdom*

* Correspondence to

Tran Thu Ngan, Ph.D.,

Wellcome Trust Fellow

Centre for Public Health, School of Medicine, Dentistry and Biomedical Sciences

Queen’s University Belfast, Belfast, BT12 6BA, United Kingdom

Email: [n.t.tran@qub.ac.uk](mailto:n.t.tran@qub.ac.uk) | Tel: +44(0)28 9097 1354

**Table S1. Distribution of cancer types by recruitment pathway**

|  | **Total**  n (%) | **Community and support-oriented**  n (%) | **Online research panel**  n (%) | **P-value** |
| --- | --- | --- | --- | --- |
| **Total** | 671 (100) | 174 (25.9) | 497 (74.1) |  |
| **ICD-10 site groups** |  |  |  |  |
| Breast | 253 (37.7) | 84 (48.3) | 169 (34.0) | <0.001 |
| Respiratory | 21 (3.1) | 13 (7.5) | 8 (1.6) |  |
| Gastrointestinal | 69 (10.3) | 27 (15.5) | 42 (8.5) |  |
| Genitourinary | 144 (21.5) | 22 (12.6) | 122 (24.5) |  |
| Skin | 52 (7.7) | 4 (2.3) | 48 (9.7) |  |
| Haematological | 71 (10.6) | 14 (8.0) | 57 (11.5) |  |
| Other solid tumours | 59 (8.8) | 9 (5.2) | 50 (10.1) |  |
| Unclear/Other | 2 (0.3) | 1 (0.6) | 1 (0.2) |  |

**Table S2. Ordered logistic regression analysis of factors associated with financial toxicity severity**

|  | Adjusted odd ratios (aOR)^§^ | 95% confidence interval |
| --- | --- | --- |
| **Recruitment pathways** |  |  |
| Online research panel^ref^ | 1.00 |  |
| Community and support-oriented | 2.33** | [1.51 – 3.62] |
| **Age group** |  |  |
| <45 years old^ref^ | 1.00 |  |
| 45-64 years old | 0.52^*^ | [0.34 - 0.80] |
| 65+ years old | 0.25^**^ | [0.13 - 0.45] |
| **Sex** |  |  |
| Male^ref^ | 1.00 |  |
| Female | 1.23 | [0.79 - 1.89] |
| **Occupation** |  |  |
| Employed full-time^ref^ | 1.00 |  |
| Employed part-time or self-employed | 1.45 | [0.91 - 2.31] |
| Unemployed/not working | 2.61^*^ | [1.28 - 5.31] |
| Retired | 0.80 | [0.40 - 1.61] |
| Disabled/too ill to work | 5.60^**^ | [3.00 – 10.43] |
| **Treatment status** |  |  |
| Completed all treatment^ref^ | 1.00 |  |
| Undergoing any type of treatment | 1.93* | [1.29 - 2.88] |
| **Personal income** |  |  |
| Less than £12,570^ref^ | 1.00 |  |
| £12,570 - £25,000 | 0.76 | [0.43 - 1.33] |
| £25,001 - £50,000 | 0.92 | [0.52 - 1.64] |
| More than £50,000 | 0.66 | [0.33 - 1.31] |
| **Stage at diagnosis** |  |  |
| Early stages^ref^ | 1.00 |  |
| Late stages | 1.13 | [0.61 - 2.67] |
| *^ref^Reference group*  *^§^Exponentiated coefficients*  *^*^p < 0.05 vs reference group; **p < 0.001 vs reference group* | | |
